# Editorial: Disorders of Circadian Rhythms

**DOI:** 10.3389/fendo.2020.00637

**Published:** 2020-09-08

**Authors:** Donají Chi-Castañeda, Arturo Ortega

**Affiliations:** Laboratorio de Neurotoxicología, Departamento de Toxicología, Centro de Investigación y de Estudios Avanzados del Instituto Politécnico Nacional, Mexico City, Mexico

**Keywords:** cancer, circadian rhythm, clock genes, metabolism, neurological diseases, shift work

Circadian rhythms are biological oscillations with a period of about 24 h, which allow the organisms to anticipate changes in the environment. These rhythms are maintained by an innate genetically determined time-keeping system called “*the molecular circadian clockwork,”* of which the suprachiasmatic nucleus (SCN) of the hypothalamus is the master biological clock and is mainly synchronized by light. The circadian system also includes peripheral clocks located in multiple cell types and tissues; these are entrained by both SCN (neural and humoral signaling) as well as other SCN-independent cues (food and temperature), resulting in a synchronized organism.

As has been established, circadian rhythmicity has a profound effect on the physiological and behavioral organization of vertebrates, so disruption of these rhythms is associated with the development of multiple clinical conditions, such as mental and metabolic diseases, cancer, addiction, and pain. In the past years it has become evident that important etiological and therapeutic connections exist between clock-based features of an organism and its pathologies. However, the functional links between disturbances of the circadian rhythms and overall health in animal models and humans are yet to be characterized.

This E-Book comprises *state-of the-art* Reviews, Original Research and Perspective contributions that feature current advancements in the molecular mechanisms and the impact of *gene-environment interactions* of circadian rhythms in diverse pathologies.

A perspective article by Nunez et al. analyzes the serious consequences of nocturnal activity in humans. In addition, the advantages and limitations of some animal models used to study these effects are discussed. Loss of circadian homeostasis is associated with pathogenesis of cancer as can be clearly understood after the critical reading of the review paper by Lin and Farkas, which is a remarkable synthesis of our current knowledge of the potential role of altered circadian rhythms in breast cancer. Discrepancies present among different studies that consider or not the rhythmicity of core clock, as well as the advantages to the use of small molecules for studying the links between circadian rhythms and cancer are also critically reviewed.

Méndez and Muñoz analyze the possible role of NADPH as a circadian and cancer-promoting metabolite. In this context, the authors focus particularly on the relationship between circadian rhythms and metabolic reprogramming (Warburg effect).

Several neurodegenerative diseases are linked with alterations in glutamate transport. Chi-Castañeda and Ortega provide a thorough review on the mechanisms of circadian regulation of glutamate transporters, including transcriptional, translational, post-translational and post-transcriptional regulation, both in neuronal and glial cells.

Light is the main synchronizer of the master clock. This oscillator encodes seasonal changes based on the amount of daylight hours (day length) and adjusts numerous biological processes. Seasonality has been documented in sleep duration, appetite, mood, social activity, among others. Garbazza and Benedetti recapitulate the current information on the relationship between biological clock and behavior, particularly mood disorders. The effects of seasonal changes of the light-dark cycle and gene polymorphisms of the core clock machinery on the behavior of patients affected by mood disorders are discussed in depth.

Disruption of circadian rhythms is also associated with reproductive problems. Caba et al. recapitulate on the essential role that the circadian clock plays in reproduction, exploiting the rabbit model that offers an extraordinary opportunity to studying this issue. In addition, they emphasize the translational importance of circadian rhythms in reproduction.

The mammalian retina contains an autonomous circadian clock that regulates diverse biochemical, cellular, and physiological processes within the eye. However, the discovery of a self-sustained oscillator in retinal pigment epithelial (RPE) cells is relatively recent, and their regulatory mechanisms are currently unknown. Therefore, using a human retinal pigment epithelium cell line, Morioka et al. studied the role that histamine signaling plays in these cells. The authors propose that the RPE oscillator is entrained by histamine *via* H_1_ receptors. In addition, the authors call the attention to the indiscriminate use of antihistaminic drugs that eventually lead to circadian rhythm disorders.

Peripheral circadian oscillators probably perform an essential role in metabolic homeostasis. Several studies have provided evidence that high sugar and/or high fat diets modify rhythmic expression of clock genes in peripheral tissues. In their article, Blancas-Velazquez et al. examined the impact of a high-energy diet on clock gene expression in different reward-related brain areas. They demonstrate that a high fat/high sugar diet affects *Per2* mRNA expression pattern in areas involved in food reward.

The pineal hormone melatonin is one of the major humoral signals from the SCN and regulates main physiological processes, such as the sleep-wake cycle, glucose, and lipid metabolism. The SCN controls melatonin synthesis and release by multisynaptic projections relaying in the superior cervical ganglia (SCG). In this sense, Mul Fedele et al. assessed the effects of SCG surgical removal on rat metabolism and diurnal rhythms of locomotor activity and feeding. Increased adipose tissue, increased body weight/food intake ratio, decreased glycemia, and increased daytime activity was found in the SCGx rats, suggesting that SCG could be altering metabolism by shifting the feeding pattern.

Circadian timing system interacts with metabolic and thermal mechanisms directly involved in the maintenance of body temperature. Accordingly, Machado et al. report cold-induced metabolic response and core clock gene expression variations in skeletal muscle (CLOCK, PER2, CRY1-2, and REV-ERBα) and brown adipose tissue (DBP and REV-ERBα) fluctuation according to the time of the day of the exposure to low temperature. Furthermore, chronic cold exposure also influences expression of genes associated in thermogenesis and substrate oxidation in a time of day and tissue-specific manner.

van der Spek et al. extensively compare clock and metabolic gene expression rhythms in mesenteric-, perirenal-, epididymal-, and subcutaneous white adipose tissue (WAT) depots. Nevertheless, no clear differences in gene expression rhythms between subcutaneous and different intra-abdominal WAT depots were found. Consequently, different WAT depots are not involved with variations in clock gene rhythmicity.

Last but not least, the review by Caba and Mendoza highlights the role of clock genes in meal anticipation. The authors present conclusive evidences demonstrating that rabbit pups are an excellent natural model to study the molecular and brain mechanism of food-anticipatory circadian behavior.

## Author Contributions

DC-C and AO have read all the contributions and wrote the Editorial Article.

## Conflict of Interest

The authors declare that the research was conducted in the absence of any commercial or financial relationships that could be construed as a potential conflict of interest.

